# Implantable cardioverter defibrillator related Actinomyces Odontolyticus endocarditis and bacteremia—First reported case

**DOI:** 10.1016/j.idcr.2021.e01228

**Published:** 2021-07-16

**Authors:** Mirai Farah Khoury, Shay Perek, Ayelet Raz-Pasteur

**Affiliations:** a Department of Internal Medicine A, Rambam Health Care Campus, Haifa, Israel; b The Rappaport Faculty of Medicine and Research Institute, Technion, Israel

**Keywords:** Actinomyces, Actinomyces Odontolyticus, Actinomyces foreign body infection, Actinomyces endocarditis

## Abstract

Actinomyces Odontolyticus is a gram-positive bacillus, usually found in the oropharynx, gastrointestinal and urogenital tracts, as commensal flora. Infections caused by this organism are rare but may occur, more commonly in immunocompromised patients. We report a case of a 54-year-old man, who presented to the emergency department (ED), with fever and chills. The patient had undergone an implantable cardioverter defibrillator (ICD) insertion, 5 months prior to his arrival, and was diagnosed with Actinomyces Odontolyticus bacteremia, during the hospitalization. Transesophageal echocardiogram (TEE) revealed vegetations on the tricuspid valve and the ICD electrode. This is the first reported case of ICD related Actinomyces endocarditis and bacteremia based on our literature review. This case emphasizes the importance of probing for the source of actinomyces infection, while considering the indolent properties of the disease.

## Highlights

• The first case of ICD related Actinomyces endocarditis and bacteremia.
• Actinomyces Odontolyticus was cultured in 6 blood samples.
• Chest scan revealed multiple acinar opacities and a cavitation.
• Transesophageal echocardiogram revealed vegetations on the tricuspid valve and the ICD electrode.

## Introduction

Actinomycosis is an indolent, slowly progressive infection caused by anaerobic or microaerophilic bacteria, primarily from the genus *Actinomyces*, which normally colonizes the mouth, large intestine, and vagina. While oral-cervicofacial manifestations are the most common, disruption of the mucosa may lead to infection of virtually any site via hematogenous spread [1]. Actinomyces Odontolyticus is an anaerobic, gram-positive bacterium, Infections with this organism are rare. Although the prognosis of the infection caused by this organism is generally favorable with medical therapy, it may lead to death if proper treatment is not initiated early [2]. Treatment involves a high-dose and prolonged course of antibiotic therapy often with Penicillin G [1]. We report a case of Actinomyces Odontolyticus bacteremia due to ICD related endocarditis and bloodstream infection, which, to our knowledge, has yet to be described in the literature.

## Case presentation

**Fig. 1:**
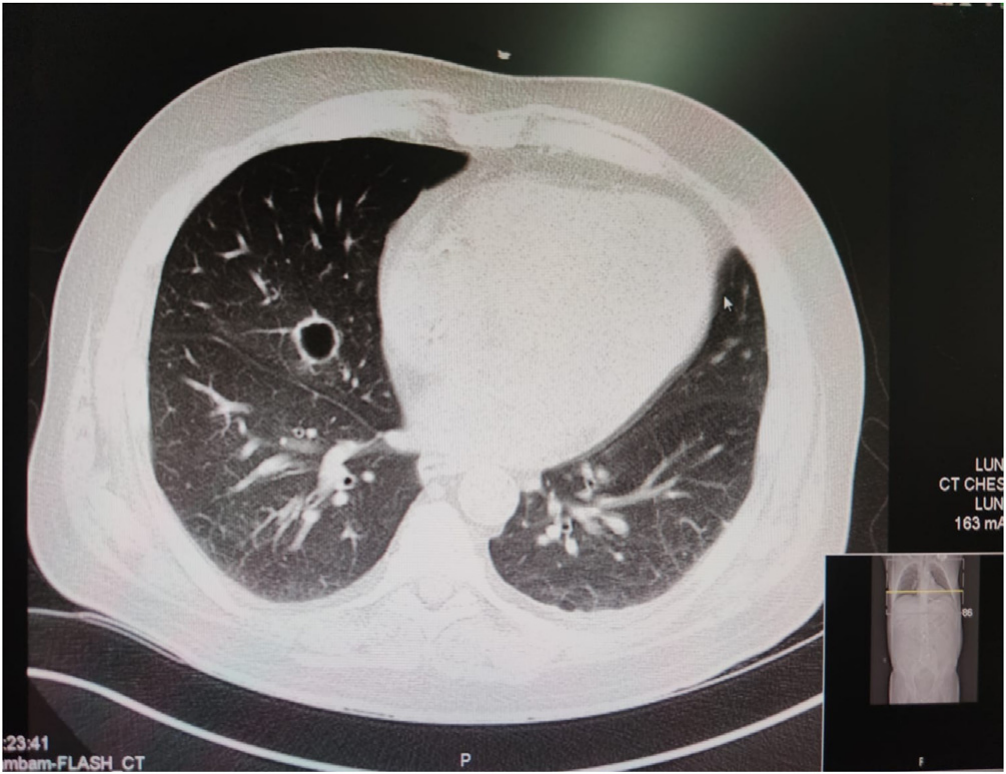
Thoracic CT scan bullous lesion suspicious for a cavitation in the right lower lobe with multiple acinar opacities.

A 54-year-old man, with a medical history of type 2 diabetes mellitus (DM), chronic kidney disease (CKD) stage 3b and heart failure with reduced ejection fraction (25 %), who had undergone prophylactic ICD insertion 5 months prior to presentation, arrived at the ED due to a two-week, persistent fever (up to 38.5 degrees Celsius), accompanied by chills and an ulcer on the tip of his toe. The patient had also complained of persistent dyspnea with a dry cough over the past several months. Upon arriving at the ED, patient`s vital signs were blood pressure 94/59 mmHg, heart rate 74 bpm, blood oxygen saturation 95 % on room air and temperature 39 degrees Celsius. Chest auscultation revealed crackles over the right lower lobe, further, an ulcer on the toe with hyperaemia, tenderness and purulence was noted. Blood tests demonstrated 12,000 leucocytes/μL (97 % neutrophils), an increase in the creatinine 4.77 mg/dL (baseline 2 mg/dL) and a Reactive C-Protein (CRP) 16 mg/dL. Blood cultures were drawn, and a COVID-19 PCR nasal swab was negative. Chest X-ray exhibited an enlarged cardiac silhouette, with no signs of pleural effusion, consolidation, or pulmonary edema. Right foot X-ray was negative for signs of deep soft tissue swelling, a periosteal reaction or cortical irregularity. Following ulcer debridement and bacterial culture swab extraction, patient was admitted to an internal medicine department with a working diagnosis of diabetic foot infection. Treatment with intravenous Amoxicillin with Clavulanic acid (1 g\12 h) and Ciprofloxacin (400 mg\ 24 h), in addition to Povidone-iodine 10 % solution foot treatment, were selected as empiric treatments. On the second day of admission, Gram staining from the ulcer swab culture resulted positive for gram-positive rods and gram-positive cocci, while blood cultures indicated gram-positive rods bacteremia. The gram-positive rods were identified as Actinomyces Odontolyticus, which were cultured in a total of 6 samples. The swab from the ulcer was positive for *Corynebacterium* Striatum and Methicillin-Susceptible Staph Aureus, but not Actinomyces. Following these results, we began a thorough workup aimed at detecting the source of the Actinomyces infection. The patient had not undergone recent oral procedures and an exam of the oral cavity was normal with no signs of infection or poor hygiene. Neck exam was negative for swelling or signs of infection. Further, the patient did not have a history of abdominal interventional procedures, or other acute gastrointestinal complaints, and abdominal exam was normal with no tenderness or palpable masses. Nonetheless, as aforementioned, the patient suffered from dyspnea and a dry cough in the past several months and during his hospitalization, with crackles on right lung auscultation, therefore another chest X-ray was performed, and showed no signs of consolidation, infiltrates, cavitation, or pleural effusion. Since this infection is more prevalent in immunocompromised patients, human immunodeficiency viruses (HIV) serology was tested and resulted negative. A computed tomography (CT) scan was performed and included the neck, chest, and abdominopelvic area. While the neck and abdominopelvic scan were normal, chest scan revealed multiple acinar opacities and a cavitation in the right lower lobe (Fig. 1) - findings suggestive of septic emboli. Due to these findings, the indolent features of the disease and the fact the patient had underwent an ICD insertion 5 months prior to the admission, we decided to perform a transesophageal echocardiogram (TEE), which revealed vegetations on the tricuspid valve and the ICD electrode. The treatment regimen was then altered to high-dose Penicillin, and the ICD was removed the following day. Electrode tip was cultured and resulted negative (which could be explained by treatment with Amoxicillin with Clavulanic acid for more than a week prior to extraction). PICC line was used for long term IV antibiotics administration. A week into his admission the patient was feeling well, afebrile, his cough was improving, CRP levels normalized, and 3 consecutive blood cultures resulted negative. The patient was released from the hospital and continued antibiotic treatment for a total of 6 weeks. 3 months later of discharge, follow up CT demonstrated full resolution of the lung disease, and no vegetations were noted on TEE. The patient remained afebrile and asymptomatic.

## Discussion

Actinomycosis is an indolent infection caused by Actinomyces and closely related species. Characteristics include chronicity, crossing of tissue boundaries, and mass-like features [1]. Infections caused by Actinomyces Odontolyticus are rarely found [3]. Aspiration is the usual source of infection in thoracic involvement of Actinomyces, in which, cavitary disease may develop and is more readily detected with CT scans, because multiple small cavities are more common than large ones [1]. While there are reports of Actinomyces Odontolyticus blood stream infection [2,4], infective endocarditis caused by Actinomyces Odontolyticus is rare with only one case out of 31 reported cases of infective endocarditis by Actinomyces species since 1939 [5]. We searched for cases of Actinomyces infection on devices and foreign bodies and found multiple reports describing this organism’s capacity to cause foreign body associated infection. Examples of such include infection of silicone tissue expanders [6], hip prosthesis [7], dialysis catheters [2], prosthetic heart valves [8], penile prosthesis [9] and ventriculoperitoneal shunts [10]. Removal of prosthetic devices and antibiotic treatment led to successful outcomes in these cases. The scarcity of reports illustrates this infection’s rarity. We did not find reports of ICD infections by Actinomyces, making this case the first reported case of ICD related Actinomyces bloodstream infection and endocarditis. In our case, we believe the most likely source of bacteremia is the ICD inserted 5 months prior. This organism can cause bacteremia, especially in immunocompromised patients like ours (DM and CKD patient), and with proper treatment, there were no clinical or radiographic signs of the disease, 3 months after admission.

## Conclusion

Actinomyces is an indolent disease that has the ability to cause infection of prosthetic devices and implants. Detection of the primary source and its eradication is the most important part of the treatment. In this case, the source of infection was an ICD electrode making it the first case reported of such infection.

## Declaration of Competing Interest

The authors report no declarations of interest.

## Funding source

No funding to declare.

## Ethical approval

Oral and written informed consent was obtained from the patient.

## Consent

A copy of the written consent is available for review by the Editor-in-Chief of this journal on request.

## Authors contribution

Mirai Farah Khoury: primary author, writing, case design, data collections.

Shay Perek: Editing, grammatical revisions.

Ayelet Raz-Pasteur: Supervising, conceived idea for the case.
